# Current insights in the development of children’s motor imagery ability

**DOI:** 10.3389/fpsyg.2015.00787

**Published:** 2015-06-10

**Authors:** Steffie Spruijt, John van der Kamp, Bert Steenbergen

**Affiliations:** ^1^Behavioural Science Institute, Radboud University NijmegenNijmegen, Netherlands; ^2^Research Institute Move, VU University AmsterdamAmsterdam, Netherlands; ^3^Institute of Human Performance, University of Hong KongHong Kong, China; ^4^School of Psychology, Australian Catholic UniversityMelbourne, Australia

**Keywords:** motor imagery, development, children, pediatric rehabilitation, mental rotation, mental chronometry

## Abstract

Over the last two decades, the number of studies on motor imagery in children has witnessed a large expansion. Most studies used the hand laterality judgment paradigm or the mental chronometry paradigm to examine motor imagery ability. The main objective of the current review is to collate these studies to provide a more comprehensive insight in children’s motor imagery development and its age of onset. Motor imagery is a form of motor cognition and aligns with forward (or predictive) models of motor control. Studying age-related differences in motor imagery ability in children therefore provides insight in underlying processes of motor development during childhood. Another motivation for studying age-related differences in motor imagery is that in order to effectively apply motor imagery training in children (with motor impairments), it is pertinent to first establish the age at which children are actually able to perform motor imagery. Overall, performance in the imagery tasks develops between 5 and 12 years of age. The age of motor imagery onset, however, remains equivocal, as some studies indicate that children of 5 to 7 years old can already enlist motor imagery in an implicit motor imagery task, whereas other studies using explicit instructions revealed that children do not use motor imagery before the age of 10. From the findings of the current study, we can conclude that motor imagery training is potentially a feasible method for pediatric rehabilitation in children from 5 years on. We suggest that younger children are most likely to benefit from motor imagery training that is presented in an implicit way. Action observation training might be a beneficial adjunct to implicit motor imagery training. From 10 years of age, more explicit forms of motor imagery training can be effectively used.

## Introduction

In a series of studies that have appeared in the last decade (Tamir et al., 2007; Lee et al., 2011; Page et al., 2011; Cho et al., 2012), it was shown that motor imagery training can be beneficial for motor rehabilitation in adult patients with acquired brain damage, in particular stroke (for reviews see Sharma et al., 2006; Dickstein and Deutsch, 2007; Zimmermann-Schlatter et al., 2008; Malouin and Richards, 2010). Motor imagery is supposed to stimulate the neural networks that underlie the planning and control of movements. As such, motor imagery training in rehabilitation is regarded as a ‘backdoor’ to facilitate a patient’s motor performance (Sharma et al., 2006).

Despite its proven effectiveness for rehabilitation in adult stroke patients, and despite converging evidence showing that problems in motor imagery are concomitant with motor control problems in congenital motor disorders such as cerebral palsy (CP) and developmental coordination disorder (DCD; Wilson et al., 2001; Crajé et al., 2010), empirical studies on motor imagery training in these children are scarce (but see Wilson et al., 2002). A likely reason for this lack of research may be that the successful application of motor imagery training necessitates that the individual has a skilled capacity to perform motor imagery. While adults were repeatedly shown to be able to use motor imagery (e.g., Cerritelli et al., 2000; Petit et al., 2003; Choudhury et al., 2007a; ter Horst et al., 2012), children’s ability for motor imagery is not very clear. The present study reviews the empirical literature on motor imagery in children to delineate the capacity of children up to 12 years of age to engage in motor imagery. The studies that were selected after a search in the literature are analyzed to provide answers to two research questions. How does motor imagery develop during childhood? At what age are children able to reliably use motor imagery? These insights are necessary to judge the feasibility of motor imagery training to promote motor performance in young children with congenital motor disorders (Steenbergen et al., 2009, 2013).

## Motor Imagery and Its Relation to Motor Performance

Probably the most influential conceptualization of motor imagery stems from Jeannerod (1994). He contended that motor imagery relates to the motor representation that is involved in the planning and execution of movements. In this view, the motor representation is a typically non-conscious process that generates or causes movements. Yet, the non-conscious motor representation can, under certain conditions, also be made conscious. Jeannerod (1994) refers to such a conscious motor representation as a motor image. “According to this definition, motor images are endowed with the same properties as those of the (corresponding) motor representation, that is, they have the same functional relationship to the imagined or represented movement and the same causal role in the generation of this movement” (Jeannerod, 1995, p.1419). Consequently, motor imagery and motor planning must be considered as functionally equivalent (Jeannerod, 1994). Motor imagery thus functions to internally simulate a future motor action without any overt motor output, i.e., the actual movement execution is inhibited (Decety and Grezes, 1999; Guillot et al., 2012). An important, but not yet fully resolved issue in this respect is the content of the motor images (and the corresponding motor representations). Most accounts conceive of a motor image as an internal model of the goal of the action that can be represented at different levels (e.g., Wolpert, 1997). These forward (or predictive) internal models contribute to volitional control by anticipating and canceling out the sensory consequences of a given movement (Vogt et al., 2013).

The link between motor imagery and motor performance is empirically supported by adult research. Neuro-imaging studies have repeatedly shown overlapping neural activity during the actual production of a movement and motor imagery of the same movement (Lacourse et al., 2005; Hanakawa et al., 2008). This includes activity in the supplementary motor areas, cerebellum, premotor cortices and the parietal cortex. For example, the parietal cortex is thought to have a role in spatiotemporal aspects of motor planning, due to its processing of perceptual information and it involves the formation of an internal model of the goal of the action (Stephan et al., 1995). In addition, patients with lesions in the parietal cortex show impaired imagery of motor tasks, as expressed by a decreased capacity to estimate the duration of the task through motor imagery (Sirigu et al., 1996, see also “Mental chronometry paradigm” below).

## Paradigms to Study Motor Imagery

The vast majority of motor imagery research uses the hand laterality paradigm and/or the mental chronometry paradigm to examine motor imagery ability in children. In the hand laterality paradigm, participants typically judge whether a displayed hand stimulus is a left or a right hand. In the mental chronometry paradigm, participants both actually perform and imagine a specific movement task. In motor imagery, a movement is imagined from a first person perspective – as if actually producing the movement oneself. Consequently, motor imagery performance is affected by the same constraints as performing an actual movement. However, participants can use alternative strategies to perform the experimental tasks within the two paradigms, for instance applying abstract rules, motor memory, or imagining the movement from a third person perspective – as if watching someone else perform the movement. These latter strategies are not constrained by, or grounded in the motor system, and hence, it will be labeled as non-motor imagery. Importantly, however, the current review focuses exclusively on the use of *motor* imagery. Hence, it is pertinent that the empirical studies allow us to demarcate the use of motor imagery and non-motor imagery strategies. The notion that only motor imagery bears a direct relation to motor planning and control processes (see also Currie and Ravenscroft, 1997) can be used to make such a distinction at a behavioral level. As we will describe below, this is indeed the case for both the hand laterality and mental chronometry paradigms.

### Hand Laterality Judgment Paradigm

**FIGURE 1:**
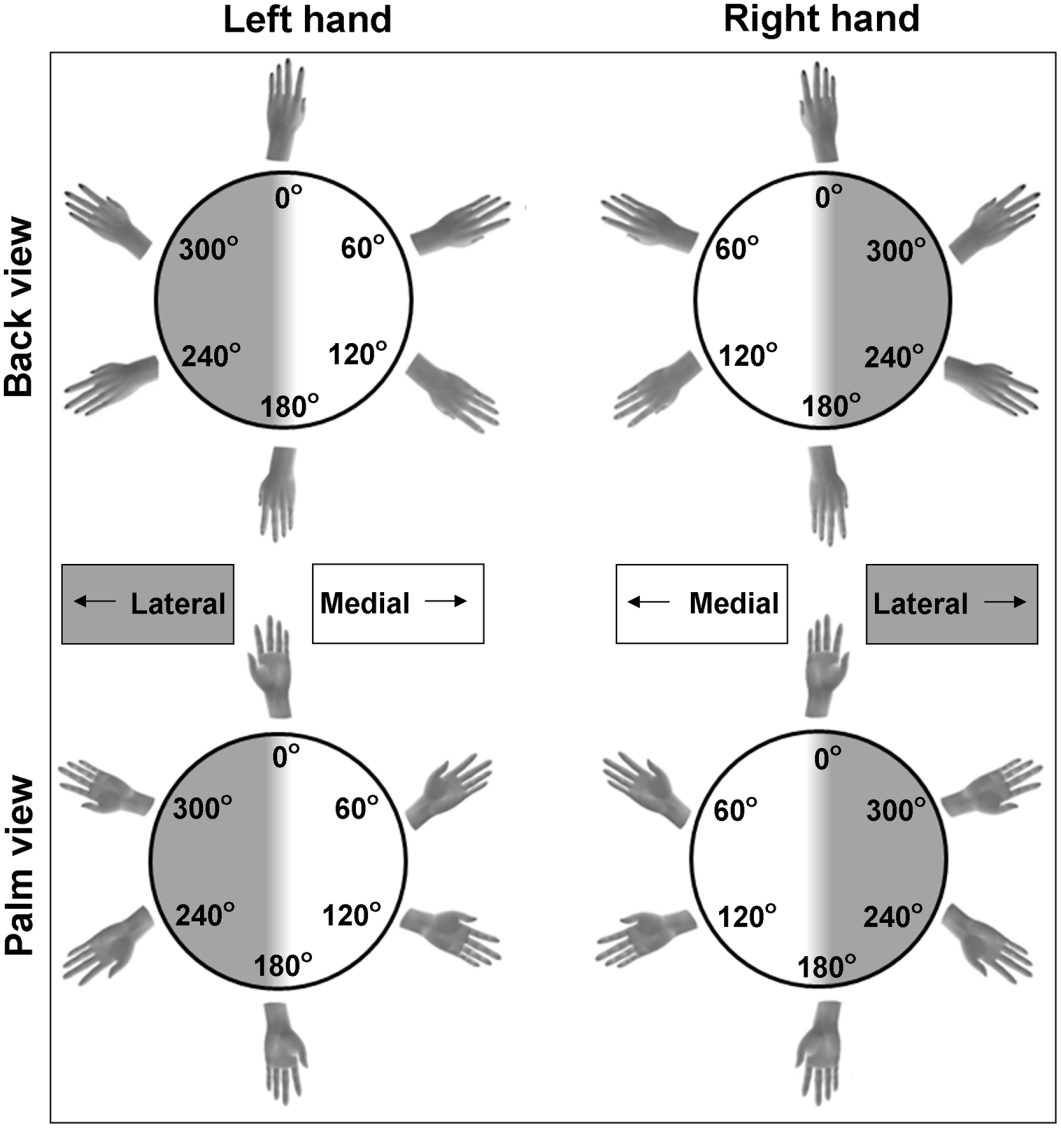
**Examples of possible stimuli for the hand laterality judgment paradigm**. Stimuli include left and right hands, commonly rotated over multiple angles of rotation and viewed from the back or the palm of the hand. Hands can be rotated to the medial side (rotation angles between 0 and 180°) and to the lateral side (rotation angles between 180 and 360°).

The first experimental paradigm that is frequently used to infer motor imagery ability is a forced-choice response task that involves hand laterality judgments. This task is a variation of classic mental rotation tasks. However, instead of judging objects, participants judge the laterality of bodily stimuli (see **Figure 1**), allowing determination of the use of motor imagery. For example, participants have to decide as quickly as possible whether the shown hand stimuli depict a left or a right hand. They do so by pressing a button that corresponds to the left or right hand, in general with their own hand palms facing down (Parsons, 1994; Shenton et al., 2004; de Lange et al., 2006; ter Horst et al., 2010). The hand stimuli are displayed in different angles of rotation (i.e., showing rotations varying between 0° with fingers pointing up to 180° with fingers pointing down) and in different directions (i.e., showing medial rotations with the fingers pointing toward the midline of the body, or lateral rotations with the fingers pointing away from the midline, see **Figure 1**). On occasions, the hands are displayed in different orientations as well (i.e., showing the back or palm of the hand, **Figure 1**).

Two outcome measures are generally analyzed: response accuracy and response duration. Response accuracy (i.e., the proportion of correct responses) is used to determine whether participants are able to solve the hand laterality judgment task above chance. Regardless of variations in response accuracy due to different rotation angles and orientations of the hand stimuli, in adults, the overall response accuracy is usually high with the proportion of correct responses rarely dropping below 90%. This indicates that adults can identify right and left hands accurately (Parsons, 1994; Shenton et al., 2004; Ionta and Blanke, 2009; ter Horst et al., 2011). Response accuracy thus provides a first indication of the ability to solve the task.

The second outcome measure is response duration, that is, the time between presentation of the hand stimulus and the button press. Commonly, only the durations of the correct responses are included for further analyses. Similar to observations of mental rotation of non-body objects (Shepard and Metzler, 1971), in adults durations vary as a function of the rotation angle of the hand stimuli. Typically, the larger the deviation from the canonical orientation (i.e., the fingers pointing up), the more time it takes to mentally rotate the hand in order to identify it as a left or right hand, at least for back view hands (Parsons, 1994; Shenton et al., 2004; de Lange et al., 2006; ter Horst et al., 2010). This pattern of response durations is taken as an indication that participants use mental rotation to solve the task. However, even though response accuracy and the effect of rotation angle on response duration are indicative for the process of mental rotation, it is critical to note that these are not sufficient to conclude that participants in fact use *motor* imagery. That is, participants can also use alternative non-motor strategies, for instance, they may apply an abstract rule or heuristic to judge hand laterality, or the rotation of the hand is imagined from a third person perspective. In sum, the use of *motor* imagery for hand laterality judgments is indicated if the pattern of response durations reflects the biomechanical constraints to which actual motor performance complies. For instance, rotating one’s own hand in a lateral rotation (away from the midline of the body) is biomechanically more difficult than rotating it to the medial side (toward the midline of the body, see **Figure 1**). Hence, increased response durations (and sometimes decreased response accuracy) when mentally rotating lateral compared to medial hand stimuli reflect the use of motor imagery. In contrast, response durations are not affected by a lateral or medial rotation direction when non-motor imagery strategies are employed. Indeed, studies in adults generally showed that lateral hand stimuli are judged slower compared to medial hand stimuli (Parsons, 1994; Shenton et al., 2004; ter Horst et al., 2011). However, the degree to which this effect is found depends on the orientation of the stimulus. This is illustrated by a larger difference in response durations between medial and lateral stimuli for palm compared to back view hands in adults (Parsons, 1994; ter Horst et al., 2010). In a similar vein, incongruence between the participant’s own hand orientation (i.e., with the back or palm side of the hand in view) and the orientation of the depicted hand results in increased response durations (and/or decreased response accuracy). The effect of own hand orientation on the response pattern was taken as evidence for motor imagery in adults (Shenton et al., 2004; de Lange et al., 2006). These behavioral indications of motor imagery in adults were confirmed at the neurophysiological level. In contrast to the employment of non-motor imagery strategies for mental rotation, brain activity during motor imagery shows substantial overlap with brain activation during actual motor performance (Dechent et al., 2004; Neuper et al., 2005; ter Horst et al., 2013).

In the hand laterality paradigm, motor imagery development is reflected by age-related increases in the degree to which the imagery performance is affected by motor constraints. We therefore considered whether previous studies found an increasing (or perhaps decreasing) effect of the medial/lateral differences on the pattern of response duration with age. To determine age of onset of motor imagery use, we evaluated the studies with respect to the age at which children’s mental rotation first display effects of motor constraints (faster responses for medial rotations and/or an effect of hand incongruence). However, before doing so, we first elaborate on the second paradigm for motor imagery, mental chronometry.

### Mental Chronometry Paradigm

The second frequently used paradigm for assessing motor imagery is mental chronometry. Here, participants are instructed to actually perform a movement task and, in a separate block or session, to imagine themselves performing the very same movement task. Mental chronometry examines whether the durations of actually performing a task and imagining the same task correspond. A high congruence between actual and imagined durations is taken as evidence for the use of motor imagery. For example, in adult participants, high correlations between the duration of actual and imagined movements were reported for goal-directed finger pointing movements (Sirigu et al., 1996; Choudhury et al., 2007b) and for goal-directed walking (Decety et al., 1989).

**FIGURE 2:**
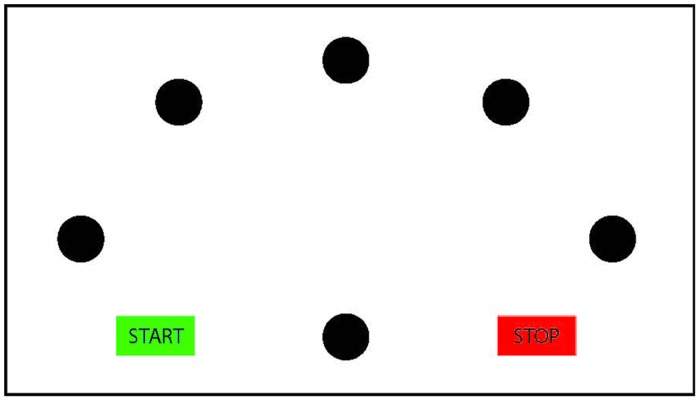
**Schematic presentation of the radial Fitts’ task**. The participants start in the green ‘start’ box, and then move to the central circle. From the central circle, they move back and forth to the five radial targets and end the movement in the ‘stop’ box. Mental chronometry studies using the Fitts’ task commonly vary target width across trials.

Importantly, however, temporal congruence may imply motor imagery; yet, non-motor imagery strategies (such as motor memory, third person perspective imagery or counting; Sharma et al., 2006; Munzert et al., 2009) cannot be automatically ruled out to account for the findings. To determine whether participants indeed employ motor imagery or instead a non-motor imagery strategy, it must also be ensured that the imagined performance is subject to the same motor constraints as the actual performance. An often-used experimental manipulation to ascertain this stems from the Fitts’ law paradigm (Fitts, 1954). Participants perform goal-directed pointing movements either repeatedly toward one target Visually Guided Pointing Task (VGPT), or consecutively toward several targets presented in a radial configuration (Virtual Radial Fitts’ Task, VRFT). The width of the target and the distance toward the target is varied across trials (for an example of a radial Fitts’ task, see **Figure 2**). Fitts (1954) described a lawful linear relation between the movement duration of pointing movements and the difficulty of the task (index of difficulty), represented by the ratio between the width of the target and the distance toward the target. Actual pointing movements adhere to this lawful relation (for a review, see Plamondon and Alimi, 1997). If participants use motor imagery in the mental chronometry paradigm, then imagined pointing should also be subject to Fitts’ law. Therefore, a linear increase in imagined duration as a function of an increasing task difficulty is an indication of the use of motor imagery. For instance, Choudhury et al. (2007a) and Cerritelli et al. (2000) have shown that Fitts’ law indeed applies for adult participants imagining visually guided pointing movements toward targets of varying width. In a similar vein, also for walking movements on paths of different length and width, adults showed compliance with Fitts’ law when mentally performing the task (Bakker et al., 2007).

In the mental chronometry paradigm, motor imagery development would be associated with an increasing congruence between the imagined and the actual task performance with age. Hence, we examined whether previous studies found evidence to support an age-related increase in temporal congruence and an increasing effect of task manipulations on the imagined task. Accordingly, the age of onset of motor imagery use would be reflected by the youngest age at which there is unambiguous evidence that children’s actual and imagined movement durations correlate and at the same time are similarly affected by task manipulations.

## Review of the Literature on Motor Imagery in Typically Developing Children

In order to establish at what age children use motor imagery, we performed a literature search on February 2nd, 2015, with a combination of the search terms ‘motor imagery’ and ‘children.’ This resulted in 54 hits in Pubmed, and 97 hits in the Web of Science search engine. Including or replacing for the search terms ‘development,’ ‘mental rotation,’ and ‘mental chronometry’ did not result in additional relevant studies, except for one article that was found when searching with the search terms “mental rotation” and “children” in Web of Science. From these studies we selected English written experimental studies that met the following two criteria: (1) the study involved a behavioral task to study motor imagery; (2) the study involved typically developing children between 5 and 12 years of age. Studies that focused on brain activation without a behavioral motor imagery task and studies that only investigated atypically developing children were excluded. The vast majority of research on motor imagery has employed the hand laterality and/or mental chronometry paradigm and they are therefore the focus of the present study. Consequently, one study that used a double-task paradigm to study motor imagery ability was excluded from further discussion (Piedimonte et al., 2014). Furthermore, studies that used a reachability paradigm to determine motor imagery ability are not used in the remainder (e.g., Gabbard et al., 2007). The rationale for excluding these studies is that it cannot be ascertained from this paradigm if the experimental tasks actually test motor imagery. Alternatively, the children may adopt an alternative non-motor imagery strategy and, for instance, merely report the perceived affordances. Review articles were also excluded. Three studies reviewed literature on motor imagery in children with motor disorders (Steenbergen et al., 2009; Gabbard and Bobbio, 2011; Adams et al., 2014) and one study reviewed literature on action representation in typically and atypically developing children (Gabbard, 2009). The literature search, however, did not return any review studies that specifically focused on the *development* of motor imagery in *typically developing* children.

The search yielded a total of 30 empirical studies that were selected for consideration (**Tables 1** and **2**). Fourteen studies focused exclusively on typically developing children, whereas the primary focus of the remaining sixteen was on children with motor disabilities, such as CP and DCD. Yet, for the present purpose it is of interest that these studies also included age-matched groups of typically developing children for comparison. These latter groups are taken into consideration. We will discuss these studies with respect to the observed age-related differences in and onset of motor imagery ability.

**Table 1:** Overview of studies that used the hand laterality judgment paradigm.

Author	Age (years)	Stimuli	Motor imagery instructions?	Considered variables	Main results
Dey et al. (2012)	5–17	Back view	No	A	No effect of age on response duration Effect of age on response accuracy
Wilson et al. (2004)	8–12	Back and palm view	No	D	Effect of rotation angle on response duration No effect of rotation angle on response accuracy
Williams et al. (2006)	7–11	Back view	Yes	D	Effect of rotation angle on response duration and response accuracy
Williams et al. (2008)	7–11	Back view	Yes	D	Effect of rotation angle on response duration and response accuracy
Caeyenberghs et al. (2009a)	7–12	Back view	Yes	D, A	Effect of rotation angle and age on response duration and response accuracy
Deconinck et al. (2009)	9	Back and palm view	No	D, B	Effect of rotation angle and biomechanical characteristics on response duration and response accuracy
Lust et al. (2006)	9–12	Back view	No	D, B	Effect of rotation angle and biomechanical characteristics on response duration
Williams et al. (2011a)	8–12	Back view	Yes	D, B	Effect of rotation angle and biomechanical characteristics on response duration
Williams et al. (2011b)	8–12	Back view	No	D, B	Effect of rotation angle and biomechanical characteristics on response duration and response accuracy
Williams et al. (2013)	7–11	Back view	No	D, B	Effect of rotation angle and biomechanical characteristics on response duration
Noten et al. (2014)	7–12	Back and palm view	Yes	C, D, B	21% of participants not above chance level Effect of rotation angle and biomechanical characteristics on response duration
Funk et al. (2005)	5–7	Back and palm view	No	C, D, B	40% of participants not above chance level Effect of rotation angle and biomechanical characteristics on response duration
Krüger and Krist (2009)	5–7	Experiment 1: Back Experiment 2: Palm	No	C, D, B, A	Experiment 1: effect of rotation angle on response duration Experiment 2: 40% of 5-year-olds not above chance level; 17% of 7-year-olds not above chance level Effect of rotation angle, biomechanical characteristics and age on response duration Effect of rotation angle and age on response accuracy
Toussaint et al. (2013)	6 and 8	Back view	No	D, B, A	Effect of rotation angle, biomechanical characteristics and age on response duration and response accuracy
Conson et al. (2013)	11–18	Back and palm view	No	D, B, A	Effect of rotation angle, biomechanical characteristics and age on response duration
Butson et al. (2014)	5–12	Back and palm view	Yes	C, D, B, A	20% of participants not above 50% response accuracy Effect of rotation angle, biomechanical characteristics and age on response accuracy and response duration

*^#^The variables that were considered in the studies are indicated by: C, response accuracy above chance; D, rotation angle; B, biomechanical constraints; A, age.*

*^∗^The study’s primary focus is on motor imagery in motor disabled children. Here we only present the results for the typically developing control group.*

**Table 2:** Overview of studies that used the mental chronometry paradigm.

Author (task)	Age (years)	Task	Considered variables^#^	Main results
Iosa et al. (2014)^∗^	4–14	Goal-directed walking	T	Effect of condition on movement duration No correlation movement durations
Molina et al. (2008)	5 and 7	Goal-directed walking	T, A	Correlation movement durations: effect of age
Skoura et al. (2009)	6–10	Drawing a maze	T, A	Effect of age and condition on movement duration Correlation movement durations: effect of age
Hoyek et al. (2009)	7–12	Obstacle course	T, A	Effect of age and condition on movement duration Correlation movement durations: effect of age
Gabbard et al. (2011)	7–11	Sequential finger movements	T, A	Effect of age and condition on movement duration Correlation movement durations for the 7- and 9-year-olds
Crognier et al. (2013)	9–21	VGPT	T, B, A	Effect of age and condition on movement duration Movement durations affected by task constraints: effect of age and condition
Maruff et al. (1999)^∗^	9–11	VGPT	T, B	Movement durations according to Fitts’ law, effect of condition Correlation movement durations
Wilson et al. (2001)^∗^	8–11	VGPT	T, B	Movement durations according to Fitts’ law, no effect of condition Correlation movement durations
Lewis et al. (2008)^∗^	8–12	VGPT	B	Movement durations according to Fitts’ law, effect of condition
Caeyenberghs et al. (2009c)	6–16	VRFT	T, B, A	Movement durations according to Fitts’ law: effect of age and condition Correlation movement durations: effect of age
Caeyenberghs et al. (2009a)	7–12	VRFT	T, B, A	Movement durations according to Fitts’ law, effect of age and condition Correlation movement durations: effect of age
Caeyenberghs et al. (2009b)^∗^	5–16	VRFT	T, B	Movement durations according to Fitts’ law, effect of condition Correlation movement durations
Williams et al. (2012)^∗^	8–12	VGPT	B	Movement durations according to Fitts’ law
Williams et al. (2013)^∗^	7–11	VGPT	T, B	Movement durations according to Fitts’ law, effect of condition Correlation movement durations
Smits-Engelsman and Wilson (2012)	5–29	VRFT	T, B, A	Movement durations: effect of age, Index of Difficulty and condition. Correlation movement durations: effect of age

*VRFT, Virtual Radial Fitts Task: 5 radial targets; VGPT, Visually Guided Pointing Task: repeated movements to 1 target; Condition, imagery vs. actual movement.*

^#^*The variables that were considered in the studies are indicated by: T, temporal congruence; B, task constraints; A, age.*

*^∗^The study’s primary focus is on motor imagery in motor disabled children. Here we only present the results for the typically developing control group.*

### The Hand Laterality Judgment Paradigm in Children

**Table 1** presents 16 studies that employed the hand laterality judgment paradigm. It is evident that nearly all studies examined the relation between response duration and angle of rotation of the depicted hands for the total group of children. A consistent finding was an effect of rotation angle on response duration, indicating increased durations as the rotation angle of the depicted hands increased. Several studies reported this relationship for children between 5 and 12 years of age (Wilson et al., 2004; Funk et al., 2005; Williams et al., 2006, 2008; Caeyenberghs et al., 2009a; Krüger and Krist, 2009). The overall observed response accuracy was found to exceed 70% in studies with children older than 7 years of age. In 5- to 7-year-olds, over half (i.e., 60%) of the children performed above chance when judging back and palm view hands (Funk et al., 2005; Krüger and Krist, 2009). These collective results suggest that the majority of children between 5 and 12 years of age are capable of mentally rotating hands, as was previously shown for non-body part objects (e.g., Marmor, 1975; Frick et al., 2013).

In addition to reporting an effect of rotation angle, several studies also assessed the impact of biomechanical constraints on total group response duration and/or accuracy via a comparison of responses to laterally and medially rotated hands. All studies that did examine the medial-lateral difference (Funk et al., 2005; Lust et al., 2006; Krüger and Krist, 2009; Williams et al., 2011a,b, 2013; Toussaint et al., 2013; Noten et al., 2014) found increased response durations for hands in lateral rotations relative to medial rotations, even when only back view stimuli were included in the study (Lust et al., 2006; Williams et al., 2011a,b, 2013; Toussaint et al., 2013). This indicates that imagery in children from 5 to 12 years of age is grounded in motor processes. An exemplary study with respect to determining the effects of biomechanical constraints was performed by Deconinck et al. (2009). They demonstrated that among 9-year-olds, biomechanical constraints affected laterality judgments in two ways. First, the judgments for laterally rotated back and palm view hands resulted in longer response durations (and were slightly, but significantly less accurate) compared to hands in medial rotations, while mental rotation of letters was not affected by medial or lateral rotations. Second, it was also found that hand orientation of the participant (i.e., with the palm up or down) relative to the orientation of the depicted hand influenced response durations (but not accuracy). Thus, response durations increased when the orientation of the participant’s and depicted hand were incongruent compared to when hand orientations were congruent. Similar results were reported for 5- to 7-year-old children that accurately performed the task (Funk et al., 2005). Taken together, the studies indicate that 5- to 12-year-old children employed the motor imagery strategy to judge hand laterality.

Besides examining overall motor imagery ability for groups of children within a certain age range, several studies have also addressed age-related differences in motor imagery in children. For instance, Caeyenberghs et al. (2009a) compared back view hand laterality judgment performance of 7- and 8-year-olds, 9- and 10-year-olds, and 11- and 12-year-olds. They found that overall the younger children responded slower and less accurate than older children, but no interaction between rotation angle and age was found. This suggests that children used the same strategy across age. Funk et al. (2005) compared the performance of 5- to 7-year-old children with the performance of adults (back and palm view stimuli). They concluded that the impact of biomechanical constraints and hand posture on laterality judgments was enhanced in the children relative to adults. They report that “these results […] strongly suggest that young children’s kinetic imagery is guided by motor processes, *even more so than in adults*” (Funk et al., 2005). Krüger and Krist (2009) and Toussaint et al. (2013) challenged this claim as biomechanical constraints had stronger effect in 8-year-olds than in 6-year-olds (back view; Toussaint et al., 2013) and when comparing 7-year-olds and adults to 5-year-olds (palm view; Krüger and Krist, 2009). In the latter study, it was concluded that “there was no indication of a particular strong link between sensorimotor and imagery processes in kindergartners [i.e., 5-year-olds]; rather, the contrary appeared true.” (Krüger and Krist, 2009). Similarly, Conson et al. (2013) also indicated that motor involvement was more pronounced in older participants, when comparing 11- and 12-year-olds to 14- and 15-year-olds and 17- and 18-year-olds. Surprisingly, they did not find a significant effect of biomechanical constraints on laterality judgments for back and palm view stimuli in 11- and 12-year-olds. Finally, Butson et al. (2014) also determined whether hand laterality judgment performance varied across age in 5- to 11-year-olds. Most 5- and 6-year-old children were not yet able to perform the task accurately above 50% chance level for back view stimuli. Response accuracy increased with age in 7- to 11-year-olds. Biomechanical constraints were only found to affect response durations for back and palm view stimuli in the 8-, 9- and 11-year-olds, but not in the 7- and 10-year-olds.

Taken together, the studies on age-related differences in motor imagery indicate that children’s ability to accurately perform the task (response accuracy) increases with age. However, there are some inconsistencies concerning the ability of 5- to 7-year-old children to accurately perform hand laterality judgments. Butson et al. (2014) reported that most children of 5 and 6 year-old were not able to accurately perform the task, while Funk et al. (2005) and Krüger and Krist (2009) and showed that only 40% of the 5- to 7-year-olds performed below chance. Most studies reported age differences on motor involvement, indicating that the use of motor imagery develops across age. Importantly, the reported age-related differences in the use of motor imagery vary across studies. Funk et al. (2005) suggested that motor involvement decreases with age. This suggests that children are more involved in the motor imagery strategy, while other strategies to solve the task (i.e., non-motor imagery) are increasingly enlisted in the task at a later age. If true, then this may accord well with one of the main tenets of Piagetian theory that the development of cognitive abilities is constructed from sensorimotor processes. That is, Piaget (1954) described that after cognitive processes emerge from the motor system, the role of motor processes in cognitive development decreases. In contrast, other studies showed an increase in motor involvement for older participants (Krüger and Krist, 2009; Conson et al., 2013; Toussaint et al., 2013). Moreover, the results of Butson et al. (2014) are inconclusive about whether the age effects reflect an increase or decrease of motor involvement with age, as the 7- and 10-year-olds showed no motor involvement, while 8-, 9-, and 11-year-olds did. Therefore, currently no definite conclusions can be drawn from studies using the hand laterality judgment paradigm about the exact development and age of onset of motor imagery between 5 and 12 years of age.

### The Mental Chronometry Paradigm in Children

The literature search yielded fifteen studies that used the mental chronometry paradigm in children (**Table 2**). Iosa et al. (2014) studied actual and imagined goal-directed walking in a small group of 4- to 14-year-olds (*n* = 8). They did not find a significant correlation between the actual and imagined durations. Molina et al. (2008) had 5- to 7-year-old children walk and imagine walking toward a target. The correlation between movement duration of the two tasks was significant for the 7-year-olds but not for their younger peers. In the study by Skoura et al. (2009), a maze drawing task was performed by children aged 6–10. Children of 6 and 8 years did not differ with respect to temporal congruence between actual performance and imagined performance, but the 10-year-olds showed significantly higher temporal congruence than the 8-year-olds. Hoyek et al. (2009) employed an obstacle course task to study mental chronometry. Temporal congruence in the 11- and 12-year-olds was significantly higher than in 7- and 8-year-old children. Finally, Gabbard et al. (2011) used a sequential finger movement task in 7-, 9-, and 11-year-old children. Both actual and imagined movement duration increased for longer sequences. In contrast to age-related differences in the other studies, Gabbard et al. (2011) reported significant correlations between movement and imagined durations only for the two *younger* groups. Collectively, these studies indicate that temporal congruence between actual and imagined performance increases from 5 to 12 years.

Still, the finding of temporal congruence in itself cannot unambiguously indicate that motor imagery is used, because participants may also have used alternative non-motor imagery strategies or even counting to solve the task. To confirm that the task was actually solved using motor imagery, 10 out of 15 studies additionally tested whether task manipulations affected the actual task and the imagined task in a similar fashion. They did so by using a paradigm based on Fitts’ law (see **Table 2**). Lewis et al. (2008) and Williams et al. (2012) used the VGPT in which the 8- to 12-year-old children made repeated pointing movements to a target. Task difficulty was systematically manipulated by varying target width. They found that for the group as a whole, durations of the actual as well as the imagined movements adhered to Fitts’ law. Four studies that included children between 5 and 16 years of age reported both temporal congruence between the two tasks and compliance with Fitts’ law for both tasks on a group level (Maruff et al., 1999; Wilson et al., 2001; Caeyenberghs et al., 2009b; Williams et al., 2013). Together, these studies showed that 5- to 12-year-olds as a group use motor imagery in a mental chronometry paradigm. However, they do not allow us to draw conclusions about onset or development, because they did not directly compare children of different ages.

Other studies extended the work on motor imagery by focusing on the age-related differences in motor imagery. First, Caeyenberghs et al. (2009c) reported that in groups of children between 6 and 16 years old, temporal congruence significantly increases with age. With respect to Fitts’ law, it was found that for the actual movement task, there was good linear fit between duration and index of difficulty for all age groups. Still, the linear fit in the imagery task increased with age; the 6- to 7-year-olds showed weaker fit than the 10- to 16-year-olds. Second, Caeyenberghs et al. (2009a) reported similar significant age-related increases in temporal congruence for 7- to 8-, and 9- to 12-year-old children. In addition, the linear fit between movement duration and index of difficulty for both tasks combined was weaker for the 7- and 8-year-olds than for the 9- and 10-, and 11- and 12-year-old children. Third, Smits-Engelsman and Wilson (2012) examined performance in the mental chronometry paradigm in participants from 5 to 29 years. It was evident that temporal congruence for the younger participants (5–7 and 8–10 years) was significantly lower than for the older participants. Although index of difficulty affected actual movement durations in all age groups, a comparable effect was absent for the imagined movement durations in children below 10 years of age. This suggests that the younger children did not use motor imagery to perform the imagery task. This result is in line with a fourth study that addressed age differences (Crognier et al., 2013). Crognier et al. (2013) tested whether manipulating task constraints for a pointing task (high vs. low inertia) would similarly affect the motor and imagined task. In contrast to performance in the motor task, the imagined task was not affected by task constraints in 9- and 11-year-olds, indicating that they did not employ motor imagery to perform the task, whereas 14- and 21-year-olds were found to employ motor imagery.

In sum, the findings from the mental chronometry paradigm indicates that children’s ability to enlist motor imagery develops until at least 12 years of age as attested by age-related increases in temporal congruence and compliance with Fitts’ law for the imagined task. The results of these studies, however, beg the question as to whether motor imagery occurs in children younger than 10–11 years of age, or whether younger children use alternative non-motor strategies to solve the task.

## The Development of Motor Imagery in Children

In the past two decades numerous studies have been performed on motor imagery in typically developing children. Most studies examined overall motor imagery ability in groups of children within a certain, often relatively large, age range. Nonetheless, some studies also have directly compared motor imagery ability between groups of children of different age. The current study is the first to provide an overview of studies on motor imagery ability in typically developing children, with a special focus on age-related differences and delineating the age at which children can reliably invoke motor imagery. Obtaining more insight in the age-related ability of children to enlist motor imagery is important for implementing motor imagery training in pediatric rehabilitation.

The current review focused on determining how motor imagery ability develops with age. Studies using the mental chronometry paradigm reported that the contribution of motor imagery becomes more salient between 5 and 12 years of age (i.e., the imagery condition more strongly complies with Fitts’ law; Caeyenberghs et al., 2009a,c; Smits-Engelsman and Wilson, 2012). There is considerable consensus from studies employing the hand laterality paradigm that from 5 to 12 years of age, children become more accurate and faster in solving the task (Caeyenberghs et al., 2009a; Krüger and Krist, 2009; Butson et al., 2014). Importantly, however, it does not necessarily follow from the enhanced ability to successfully perform the task that with development children do actually use motor imagery more or become more proficient in using motor imagery. Alternative strategies, such as for instance non-motor imagery (i.e., with a third rather than first person perspective) may also increasingly contribute to solving the mental imagery tasks successfully. In line with studies using the mental chronometry paradigm, most studies using the hand laterality paradigm also reported that motor involvement increased with age (Krüger and Krist, 2009; Conson et al., 2013; Toussaint et al., 2013), albeit at younger ages than in mental chronometry studies. Nonetheless, Funk et al. (2005) contradict the evidence for an increasing role of motor imagery with age, by showing that for 5- to 7-year-old children who accurately performed the task, hand laterality judgments are fully grounded in motor processes, while later in development the contribution of motor processes decreases (Funk et al., 2005). It is difficult to explain the deviating results of Funk et al. (2005) based on differences in the experimental set-up. Although studies show methodological differences, for instance with regard to inclusion of back and/or palm view hand stimuli and specific first person perspective motor imagery instructions (**Table 1**), these differences do not seem to provide a systematic explanation for the discrepant findings. For instance, even though the stimulus set of Funk et al. (2005) and Conson et al. (2013) both included back and palm view stimuli and no specific motor imagery instructions were provided, Funk et al. (2005) report a decrease of biomechanical constraints in imagery performance with age, whereas Conson et al. (2013) showed the opposite effect, that is, an increased effect of biomechanical constraints (see also Krüger and Krist, 2009; Toussaint et al., 2013). Taken together, most studies indicate that motor imagery increases with age, but it is difficult to draw definite conclusions on the exact developmental trajectory. In this respect, future work on motor imagery development would surely benefit from a longitudinal design, which remarkably, has never been adopted thus far.

With respect to the age at which children start to use motor imagery, studies employing the hand laterality judgment paradigm reported that little over half of 5- to 7-year-old children are already capable of using motor imagery to accurately perform the task including palm view stimuli (Funk et al., 2005; Krüger and Krist, 2009). Butson et al. (2014), however, could not confirm these observations, and reported that only a few of the 5- to 6-year-olds were able to accurately perform the task. The latter finding is surprising, as Butson et al. (2014) used hand stimuli that were relatively easy to judge (back view, with the fingers pointing up) to determine whether children were able to judge hand laterality. Moreover, they reported that 7-year-olds did not appear to enlist motor imagery reliably. In fact, Conson et al. (2013) argued that even children as old as 11 or 12 years did not use a motor imagery strategy – considering other reports for the hand laterality paradigm, this study is clearly an exception. Conson et al. (2013) included palm view stimuli, that were previously reported to induce the effect of biomechanical constraints on adult’s task performance, compared to back view performance (see Parsons, 1994; ter Horst et al., 2010). The orientation of the stimuli can therefore not account for the absence of motor imagery indications. Furthermore, a lack of specific task instructions could not explain the absence of motor imagery reported by Conson et al. (2013) as also Funk et al. (2005) and Krüger and Krist (2009) did not provide specific task instructions. Taken together, these studies suggest that differences in methodological set-up between studies cannot easily account for the differences in task performances between the studies. However, a late emergence of motor imagery is in line with observations in the mental chronometry paradigm (Smits-Engelsman and Wilson, 2012), which indicate that the use of motor imagery does not emerge before 10–12 years of age. Only very few children of 5–7 years of age have been shown to be capable of using motor imagery on the mental chronometry paradigm (Caeyenberghs et al., 2009c; Smits-Engelsman and Wilson, 2012).

In sum, previous motor imagery studies suggest that possibly a small proportion of the 5-year-olds is able to accurately use motor imagery. Yet the evidence is equivocal, and hence the exact age of onset of motor imagery use for both paradigms remains to be verified. Clearly, there are significant individual and task differences in young children’s motor imagery. These inter-individual differences in motor imagery ability might be explained by cognitive and motor abilities that can facilitate or constrain motor imagery development, such as executive functioning (e.g., working memory, inhibition, attention; see also Krüger and Krist, 2009), motor planning ability, movement experience (see also Caeyenberghs et al., 2009a) and IQ. For example, working memory has been suggested to be related to motor imagery ability in adults (Malouin et al., 2004; Choudhury et al., 2007a; Gabbard et al., 2013). The rapid development of executive functions during childhood (i.e., Brocki and Bohlin, 2004) might therefore be tightly coupled to motor imagery development. Challenges for future studies remain to determine factors such as working memory that might impact children’s motor imagery development. In doing so, we recommend to first establish whether individual participants use motor imagery and then compare the children that do successfully use motor imagery to the children that are not using motor imagery.

The hand laterality judgment paradigm and the mental chronometry paradigm are commonly used measures of motor imagery in adults (as described in the review of Munzert et al., 2009). From the overview of the literature on age-related differences in children’s motor imagery ability it is evident, however, that the results commonly differ between these two imagery tasks. Most hand laterality judgment studies suggest that a considerable number of 5- to 8-year-olds and nearly all older children are able to use motor imagery (Funk et al., 2005; Caeyenberghs et al., 2009a; Krüger and Krist, 2009; Toussaint et al., 2013). By contrast, for the mental chronometry paradigm it is estimated that only one out of ten 5- to 7-year-old children use motor imagery, while only after 10 years of age all children do so (Smits-Engelsman and Wilson, 2012). Obviously, the discrepant developmental patterns may arise from distinct task characteristics that hamper the expression of motor imagery ability more during mental chronometry than during judgment of hand laterality. A likely explanation may be sought in the nature of the paradigms, invoking motor imagery either explicitly or implicitly. In the mental chronometry paradigm children are often made aware and instructed to use motor imagery explicitly, whereas the hand laterality judgment paradigm is more implicit and instructions regarding motor imagery are often lacking (cf. Caeyenberghs et al., 2009a). Previous studies showed that implicit learning (i.e., without instructions that make children aware of what they have to learn or do) is relatively unaffected by age, whereas explicit learning shows clear increases with age (Meulemans et al., 1998; Vinter and Detable, 2008). Accordingly, in young children motor imagery may be more easily induced without instructions that make children aware of what they have to do, while explicit instructions to employ motor imagery may actually hinder its use, especially at a younger age. In line with this suggestion, the present results show that young children already used motor imagery in a task with implicit instructions (hand laterality judgment paradigm), but did not use motor imagery in a task with explicit instructions at a young age (mental chronometry paradigm).

In addition to an extensive body of literature supporting the beneficial effects of incorporating motor imagery training in standard rehabilitation protocols in adults (i.e., Malouin and Richards, 2010), two studies in children in the age range of 7–12 years old underline the potential of motor imagery training in children (Wilson et al., 2002; Doussoulin and Rehbein, 2011). However, prior to a systematic and effective application of motor imagery training in pediatric rehabilitation, knowing from what age and under what pre-conditions children are able to enlist motor imagery is of utmost importance. In this respect, the current review suggests that children as young as 5 years can enlist motor imagery in an implicit way, while explicitly adopting motor imagery might not be possible before 10 years of age. Obviously, if confirmed, then this is particularly relevant for developing age-related content of motor imagery training programs. With respect to implicit motor imagery training for the youngest children, an interesting adjunct may be offered by action observation training. Motor imagery and action observation substantially overlap in terms of their neuro-anatomical basis (Grezes and Decety, 2001; Filimon et al., 2007). This commonality may provide a promising avenue for stimulating the networks involved in motor control and development (Buccino et al., 2006). For example, a recent study on action observation in addition to actually performing movements showed clear benefits of action observation for motor performance in 6–11-year-old children with CP (Buccino et al., 2012). Contrary to children who watched videos without motor content, children who were watching videos of others producing actions led to an increase in motor function. Accordingly, action observation may be a valuable aid to motor imagery in the very young children that cannot be instructed about using motor imagery. For children older than 10 years, more explicit forms of motor imagery training seem viable. Future research must examine whether these instructions can be as detailed as has been successfully used in motor imagery training in adults with stroke (e.g., Dijkerman et al., 2004). Subsequently, identifying factors that limit or facilitate the use of motor imagery can aid the selection of children that may benefit from implicit or explicit motor imagery training.

## Conflict of Interest Statement

The authors declare that the research was conducted in the absence of any commercial or financial relationships that could be construed as a potential conflict of interest.
